# Sepsis screening tools in resource-limited settings: a systematic review and meta-analysis of diagnostic accuracy in low- and middle-income countries

**DOI:** 10.3389/fpubh.2026.1782420

**Published:** 2026-05-20

**Authors:** Jiale Tong, Peng Deng

**Affiliations:** 1 Department of Emergency Medicine, West China Hospital, Sichuan University, Chengdu, China; 2 West China School of Nursing, Sichuan University, Chengdu, China; 3 Institute of Disaster Medicine, Sichuan University, Chengdu, China; 4 Nursing Key Laboratory of Sichuan Province, Sichuan University, Chengdu, China; 5 Department of Emergency Medicine, West China Hospital, and Disaster Medical Center, Sichuan University, Chengdu, China

**Keywords:** diagnostic accuracy, low- and middle-income countries, meta-analysis, qSOFA, screening tools, sepsis, systematic review

## Abstract

**Background:**

Sepsis causes disproportionate mortality in low- and middle-income countries (LMICs), yet evidence on screening tool performance in these resource-limited settings remains fragmented.

**Objective:**

This systematic review and meta-analysis aimed to evaluate and compare the diagnostic accuracy of sepsis screening tools—ranging from purely clinical assessments (qSOFA, NEWS, MEWS, UVA) to those incorporating laboratory parameters (SIRS, SOFA)—for sepsis identification in adult populations within LMICs.

**Methods:**

A comprehensive search of PubMed, Embase, Cochrane Library, Web of Science, and Global Index Medicus was conducted from inception through June 2025. Eligible studies evaluated sepsis screening tools ranging from purely clinical bedside assessments (qSOFA, NEWS, MEWS, UVA) to those requiring basic laboratory parameters (SIRS, SOFA), enabling comparison across the resource-availability spectrum in LMICs. Bivariate random-effects models were employed to calculate pooled sensitivity, specificity, and area under the hierarchical summary receiver operating characteristic curve (AUROC). Evidence quality was assessed using the GRADE approach.

**Results:**

Twenty-seven studies encompassing 30,310 patients across 14 LMICs were included. qSOFA demonstrated pooled sensitivity of 0.51 (95% CI: 0.42–0.60) and specificity of 0.83 (95% CI: 0.77–0.88) with AUROC of 0.74. SIRS exhibited high sensitivity (0.86) but poor specificity (0.32). NEWS achieved the highest point estimate of discriminative ability (AUROC 0.77, 95% CI: 0.73–0.81), followed by SOFA (AUROC 0.75, 95% CI: 0.71–0.79) and UVA (AUROC 0.74, 95% CI: 0.70–0.78), although confidence intervals overlapped substantially across tools. qSOFA yielded a positive likelihood ratio (LR+) of 3.00 and a negative likelihood ratio (LR−) of 0.59, indicating moderate rule-in but limited rule-out utility. Substantial heterogeneity was observed across studies (I^2^ > 75%).

**Conclusion:**

No single screening tool demonstrates optimal performance across all metrics in LMIC populations. When analysis is restricted to purely clinical tools requiring no laboratory parameters, NEWS (AUROC 0.77) and qSOFA (AUROC 0.74) demonstrate comparable discriminative ability with broadly overlapping confidence intervals, supporting their consideration in the most resource-constrained settings. However, the limited rule-out capacity of qSOFA (LR − 0.59) suggests it should not be used as a standalone screening tool. Tool selection should be guided by local healthcare priorities and available laboratory capacity, with tiered screening strategies potentially optimizing sepsis recognition while ensuring efficient resource allocation.

## 1 Introduction

Sepsis represents a life-threatening organ dysfunction caused by a dysregulated host response to infection, posing one of the most significant challenges to global public health systems (1). According to epidemiological estimates, approximately 49 million cases of sepsis occur worldwide each year, resulting in 11 million deaths, which accounts for nearly 19.7% of all global deaths (2). The pathophysiology of sepsis involves an intricate combination of pro-inflammatory and anti-inflammatory reactions that cause significant tissue damage and the possible onset of multiple organ failure if not identified and treated in a timely fashion (3). Though sepsis occurs in various societies belonging to different economic backgrounds, its impact is mainly observed in low- and middle-income nations (LMICs), which account for more than 80% of sepsis-related deaths due to high mortality rates often above 50% (4). This gap also highlights the inherent discrepancies in healthcare infrastructure, diagnosis, and treatment accessibility in developed countries compared with settings with limited resources.

The management of sepsis has evolved considerably over the past decades, with international guidelines emphasizing the critical importance of early recognition and timely intervention (5). The Surviving Sepsis Campaign has established comprehensive recommendations for sepsis management, including the administration of appropriate antimicrobial therapy within the first hour and aggressive fluid resuscitation. However, it has been observed that the Surviving Sepsis Campaign recommendations and guidelines have been made largely based on evidence from resource-rich countries that have well-organized intensive care units and laboratory facilities. It has been noted that following the Surviving Sepsis Campaign recommendations and guidelines in LMICs can pose significant challenges due to limitations in laboratory testing, intensive care units, and specialized healthcare providers (6). It can be assumed that a large population of sepsis patients in such environments is being managed in general wards and emergency care units, but not in intensive care units. Sepsis in LMICs may also have different clinical manifestations based on infection profiles that include tropical infections, malaria, tuberculosis, and HIV (7).

Early sepsis identification is not only an issue of concern within the healthcare system but also an utmost priority from the perspective of public health, especially in resource-limited settings. World Health Organization gave importance to sepsis at the international level through World Health Assembly Resolution 70.7 in 2017, which promotes improved prevention, diagnosis, and management of sepsis globally (2). Validated screening approaches with varying resource requirements—from purely clinical assessments to those incorporating basic laboratory parameters—can aid in stratified care delivery in primary healthcare systems and assist in resource allocation in the healthcare sector, which is limited in nature. The goal of achieving Universal Health Coverage and minimizing preventable mortality in areas that lack the ability to conduct in-depth diagnosis can be accomplished using the above approach (8).

In the past three decades, various sepsis screening models have been proposed, ranging from no resource utilization to a combination of various degrees of laboratory values. The Systemic Inflammatory Response Syndrome (SIRS) criteria, which were first proposed in 1992, are based on body temperature, heart rate, breathing rate, and white blood cell count. It is important to note that the inclusion of white blood cell count in this set of criteria requires laboratory investigation, making this tool one step higher on the resource utilization scale. The SOFA score, which was first proposed in 1996, is based on a six-system organ assessment and requires various laboratory values, including arterial oxygen tension, platelet count, bilirubin, and creatinine, making this tool the highest on the scale. The quick Sequential Organ Failure Assessment (qSOFA) score, which was proposed in 2016, recognizes resource constraints and relies on three simple bedside criteria to evaluate patients: altered mental status, either a respiratory rate of 22 or more per minute, and systolic blood pressure of 100 mmHg or less (3). National Early Warning Score (NEWS) has seven physiological criteria, while Universal Vital Assessment (UVA) has been specifically designed for populations in sub-Saharan Africa because of the different epidemiology found in those populations.

Despite the availability of various screening tools, there remain significant gaps in the present appreciation about the diagnostic performance of these tools among LMIC populations. Validation of various tools, as mentioned, has largely been done in settings of high-income countries, making these findings non-generalizable in resource-limited settings (7). Although various studies have examined the diagnostic validity of sepsis screening tools available for bedside usage in LMICs, a systematic synthesis of this evidence has not been established. Moreover, comparison studies among various sepsis screening tools in LMIC are very scarce, and this provides substantial challenges for healthcare practitioners who seek to make well-informed decisions on sepsis screening tool implementation based on available systematic evidence. The data variability within past studies also contributes to challenges in interpretation (8).

This systematic review and meta-analysis seeks to address the aforementioned gaps in the research by systematically comparing the diagnostic accuracy of various sepsis screening tools along the entire range of resource requirements, from purely clinical tools (qSOFA, NEWS, MEWS, UVA) to those that require laboratory parameters (SIRS, SOFA), in adult populations in LMICs. The primary aim of this systematic review is to systematically compare the sensitivity, specificity, and area under the curve of these sepsis screening tools. Secondary objectives include evaluating the performance of each model in predicting mortality, evaluating the effect of geographic location, health facility setting, and patient factors on model performance, and making evidence-based recommendations for sepsis-screening in resource-poor settings. The paper consolidates findings of 27 existing studies involving 30,310 patients in 14 LMIC countries using bivariate random effects models and hierarchical summary receiver operating curve analysis. The findings of this study are aimed at informing practice and policy, adding to the global health agenda in reducing preventable sepsis-related mortality in settings that are most impacted by sepsis.

## 2 Materials and methods

### 2.1 Protocol and search strategy

This systematic review and meta-analysis was conducted in accordance with the Preferred Reporting Items for Systematic Reviews and Meta-Analyses of Diagnostic Test Accuracy Studies (PRISMA-DTA) guidelines. This review was not prospectively registered with PROSPERO. However, a detailed written protocol specifying eligibility criteria, search strategy, data extraction procedures, quality assessment methods, and statistical analysis plans was finalized prior to the commencement of literature screening. All pre-specified primary and secondary outcomes were reported regardless of statistical significance, and no post-hoc changes were made to the eligibility criteria or analytical methods after data extraction commenced. The complete protocol is available from the corresponding author upon reasonable request.

The research team conducted an extensive literature search using a variety of electronic bibliographic databases from the inception of the databases until June 2025. The databases that were searched include MEDLINE via PubMed, Embase via Ovid, Cochrane Central Register of Controlled Trials (CENTRAL), Web of Science Core Collection, and the Global Index Medicus to search for studies from low- and middle-income countries. The research team also searched clinical trial registries and supplementary sources as detailed below.

The strategy combined MeSH heading terms with free-text keywords in four conceptual fields: (1) sepsis, severe sepsis, septic shock, and associated infectious diseases; (2) screening and diagnostic scores: qSOFA, SOFA, SIRS, NEWS, MEWS, UVA; (3) terminology for diagnostic accuracy, including the concepts for sensitivity, specificity, and the ROC curve; and (4) names of LMICs and descriptions of low-resource settings. The Boolean operators AND/OR were used to link concepts both within and between these fields, and modifications could be considered based on the properties of each database. The complete strategies for each database are shown in Supplementary Table S1.

In order to ensure that the review is comprehensive, other strategies for searching were employed. The reference lists of all included studies and relevant systematic reviews were checked for potentially eligible papers. Grey literature was also searched for by accessing ClinicalTrials.gov and the WHO International Clinical Trials Registry Platform. Global Index Medicus was considered a primary source of literature for searching, as it includes literature that is not available in mainstream databases. The authors of the included papers were contacted when additional information was needed.

### 2.2 Eligibility criteria

For this systematic review, the inclusion and exclusion criteria were identified using the PICOS (Population, Index test, Comparator, Outcomes, Study design) criteria. The population of interest included adults aged 18 years and above suspected of or proven to be suffering from sepsis, attending health facilities in low- and middle-income countries as classified by the World Bank. There were no restrictions based on sex or underlying comorbid conditions. Studies in which the population included those from high income countries and those in pediatric settings were excluded.

The index tests of interest were the sepsis screening tools that are often applied or evaluated in the context of the LMICs. These were categorized into two groups based on the resource utilization: (1) the tools that are based solely on the clinical criteria without the use of laboratory values, such as the quick Sequential Organ Failure Assessment (qSOFA), the National Early Warning Score (NEWS/NEWS2), the Modified Early Warning Score (MEWS), and the Universal Vital Assessment (UVA); and (2) the tools that incorporate basic laboratory values, including the Systemic Inflammatory Response Syndrome (SIRS) criteria that require the use of white blood cell count and the Sequential Organ Failure Assessment (SOFA) that require the use of platelet count, bilirubin, creatinine, and the PaO2/FiO2 ratio. The inclusion of these two categories is to ensure that the study is comprehensive to cover the resource availability spectrum that is often encountered in the context of the LMICs. The reference standards accepted for the study were the Sepsis-3 criteria, the clinical diagnosis as evaluated by the clinicians, or the hospital mortality as a surrogate. The key outcomes included measures for diagnostic accuracy, such as sensitivity, specificity, area under the receiver operating curves (AUROC), positive predictive value(PPV), negative predictive value(NPV), and likelihood ratios. Secondary outcomes were estimated as odds ratios based on the ability to predict mortality. The eligible study designs were prospective and retrospective cohort studies and cross-sectional studies for diagnostic accuracy. Reviews, editorials, case reports, and studies that did not report data for diagnostic accuracy were excluded. Only English-language sources were included, and there were no publication year restrictions.

### 2.3 Study selection and data extraction

The literature screening process was completed by two trained independent reviewers using the Covidence systematic review software. The screening was carried out in two stages: preliminary screening of titles and abstracts followed by full-text evaluation. Disagreements were resolved through discussion or arbitration by a third senior reviewer. The entire screening process was documented with reasons for exclusion, and a PRISMA flowchart was generated. Prior to formal screening, two reviewers independently screened 50 randomly selected documents and calculated Cohen’s kappa coefficient, with formal screening commencing only after the kappa value reached 0.8 or above.

Data extraction was performed using a pre-designed standardized form by two independent reviewers. The extracted content included study characteristics (author, year, country, design, setting), participant characteristics (sample size, age, sex, infection types), index test details (tool, threshold, timing), reference standard definition, and diagnostic accuracy data including true positive, false positive, false negative, and true negative values. For studies with incomplete data, the research team contacted original authors to obtain necessary information.

### 2.4 Risk of bias assessment

The methodological quality of included studies was assessed using the Quality Assessment of Diagnostic Accuracy Studies-2 (QUADAS-2) tool, which evaluates risk of bias across four domains: patient selection, index test, reference standard, and flow and timing. Each domain was rated as a low, high, or unclear risk of bias using the signaling questions listed within the QUADAS-2 framework. Items regarding applicability concerns were also assessed for the domains of patient selection, index test, and reference standard. The quality evaluation of the studies was done by two independent reviewers, with any inconsistencies resolved by consensus. Results are presented graphically using summary plots created by the Review Manager computer program.

### 2.5 Statistical analysis

The statistical analyses were done using the R software (version 4.3.0), utilizing the mada and metafor packages, and the Stata software (version 17.0). To calculate the pooled estimates of sensitivity and specificity for each screening tool that consisted of three or more studies, the bivariate random effects model was used. Hierarchical summary receiver operating characteristic (HSROC) curves and the corresponding area under the curve with 95% confidence intervals were employed.

The heterogeneity of the included studies was assessed by Cochran Q test and I^2^ statistics, where I^2^ values of less than 25%, between 25 and 75%, and higher than 75% were regarded as low, moderate, and high heterogeneity, respectively. Due to the expected heterogeneity in terms of clinical and methodological diversity of the anticipated results of the diagnostic accuracy studies conducted in multiple LMICs, the bivariate random-effects model was chosen *a priori* to address the anticipated heterogeneity between the included studies. Several *a priori* planned methods were used to address the sources of heterogeneity. The subgroup analyses were a priori planned by type of screening aid, geographic region (South Asia, Southeast Asia, Sub-Saharan Africa), continental cohort (Africa vs. Asian), type of clinical setting (emergency department, intensive care unit, general ward), and baseline mortality rate. The meta-regression method was used to evaluate the continent (Africa vs. Asia) as a potential predictor of diagnostic accuracy. Sensitivity analyses were performed by excluding each study in turn and by excluding studies at high risk of bias.

A further pre-specified sensitivity analysis was conducted by only using screening tools that do not require laboratory parameters (qSOFA, NEWS, MEWS, UVA), in order to determine whether or not using screening tools that do require laboratory parameters (SIRS, SOFA) impacted the overall results. The publication bias was checked using Deeks’ test for funnel plot asymmetry, where there were ten or more studies included. The certainty of evidence was assessed using the Grading of Recommendations Assessment, Development and Evaluation (GRADE) approach (45), considering risk of bias, indirectness, inconsistency, imprecision, and publication bias. All statistical tests were two-sided with significance level set at *α* = 0.05.

## 3 Results

### 3.1 Literature search and study selection

A systematic search of the five electronic databases yielded 2,156 potentially eligible records. After removal of the 487 identified duplicates, 1,669 citations underwent the title and abstract screening. Among the initially screened citations, 1,528 were excluded because they did not meet the pre-specified inclusion criteria and comprised a large number of irrelevant and non-human studies, editorials, and commentaries. The next phase involved the evaluation of the full texts of the remaining 141 articles to check the eligibility standards.

During extensive full text screening, 114 articles were removed for the following reasons: 47 studies took place in high-income country settings alone; 28 studies had inadequate diagnostic accuracy data available for the formation of two-by-two contingency tables, or for calculations for sensitivity and specificity; 19 studies had predominantly pediatric or mixed study populations, without analyzing data in adults separately; 12 studies had study designs unsuitable for obtaining diagnostic accuracy parameters, such as in case reports or narrative reviews; and 8 studies assessed screening tests beyond the inclusion criteria. No additional articles were found in either reference screening or grey literature searches.

**Figure 1:**
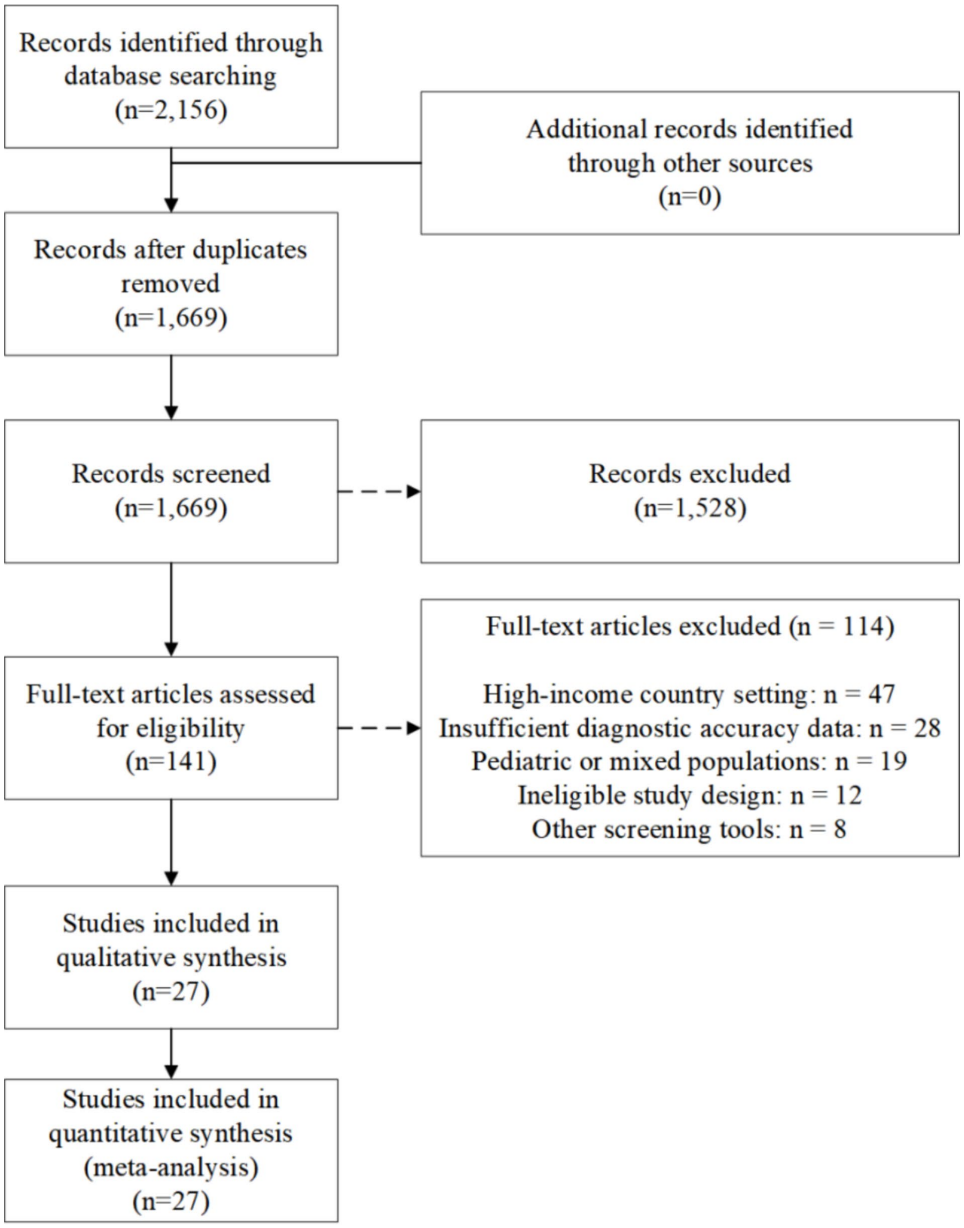
PRISMA flow diagram of study selection process.

In the end, 27 studies met all the selection criteria and were thus included in the qualitative synthesis and meta-analysis. A total of 30,310 suspected or confirmed sepsis cases among adults from 14 low- and middle-income countries participated in studies included in this review, published between 2015 and 2025. The studies included evaluated the accuracy of various screening methods for sepsis in the clinical setting. These studies include 22 studies for qSOFA, 11 studies for SOFA, 7 studies for the SIRS criteria, 5 studies for the NEWS scale, and 4 studies for the UVA scale. The flow of the full study selection in line with the PRISMA-DTA is shown in Figure 1.

### 3.2 Characteristics of included studies

The 27 studies included in this analysis were published between 2015 and 2025 and included a cumulative 30,310 adult participants who were suspected of or diagnosed with sepsis in 14 low- and middle-income countries. The studies are described in more detail in Table 1. The geographic distribution demonstrated substantial representation from three major regions. South Asia provided 10 studies (37.0%), with contributions from India (*n* = 6), Pakistan (*n* = 2), Nepal (*n* = 1), and Sri Lanka (*n* = 1). Sub-Saharan Africa also provided 10 studies (37.0%), with contributions from Uganda, Tanzania, Ethiopia, Malawi, and Rwanda, including one multi-site study from six countries in sub-Saharan Africa. Southeast Asia provided 5 studies (18.5%), with contributions from Indonesia, Vietnam, and Thailand. The remaining 2 studies (7.4%) were multi-regional studies conducted across several LMIC countries, including sites in Ghana and Cambodia.

**Table 1:** Characteristics of included studies (*n* = 27).

Study, year	Country	Region	Sample size	Study design	Setting	Screening tools	Primary outcomes
Asiimwe et al. (18), 2015	Uganda	Sub-Saharan Africa	317	Prospective cohort	ED	Simple prognostic index, MEWS	Simple index≥3 mortality OR 3.4 (95% CI 1.6–7.3); OR 2.3 in validation
Baig et al. (19), 2018	Pakistan	South Asia	760	Prospective cohort	ED	qSOFA, SOFA	qSOFA severe sepsis AUROC 0.92 (95% CI 0.89–0.94), sensitivity 96%, specificity 87%; septic shock AUROC 0.89
Blair et al. (20), 2023	Ghana, Cambodia, USA	Multi-region	567	Prospective cohort	ED/Ward	qSOFA, MEWS, NEWS, UVA, SIRS	UVA C-statistic 0.73 (95% CI 0.69–0.78); qSOFA 0.70; NEWS 0.68; MEWS 0.63
Boillat-Blanco et al. (21), 2018	Tanzania	Sub-Saharan Africa	519	Prospective cohort	ED	qSOFA, SOFA, SIRS	qSOFA AUROC 0.80 (95% CI 0.73–0.87); SOFA AUROC 0.79; SIRS AUROC 0.61
Carugati et al. (22), 2018	Tanzania	Sub-Saharan Africa	419	Prospective cohort	ED	MEWS, qSOFA, IMAI	IMAI emergency signs good accuracy; qSOFA≥2 associated with mortality
Dat et al. (23), 2018	Vietnam	Southeast Asia	393	Retrospective cohort	ED/ICU	SOFA, qSOFA	SOFA AUROC 0.858 (ICU), 0.667 (ward); qSOFA AUROC 0.692 (ICU), 0.527 (ward)
Fuchs et al. (24), 2021	Ethiopia	Sub-Saharan Africa	170	Prospective cohort	ED/Ward	SOFA, qSOFA	qSOFA sensitivity 54.3%, specificity 66.7%; mortality 29.4%
Ghimire et al. (25), 2020	Nepal	South Asia	148	Prospective cohort	ED	RDW, APACHE II, SOFA	RDW AUROC 0.734; APACHE II AUROC 0.728; SOFA AUROC 0.680
Gunawan et al. (26), 2024	Indonesia	Southeast Asia	150	Retrospective cohort	ICU	qSOFA, mROX	qSOFA≥2 OR 3.69; mROX≤3.20 OR 21.50; combined AUROC 0.791
Huson et al. (27), 2017	Malawi	Sub-Saharan Africa	324	Prospective cohort	ED	qSOFA	qSOFA AUROC 0.73 (95% CI 0.68–0.78); AUROC 0.77 with altered mental status
Kayambankadzanja et al. (28), 2020	Malawi	Sub-Saharan Africa	1,135	Cross-sectional	ED/Ward	qSOFA	qSOFA≥2 sensitivity 31.8%, specificity 82.1%; mortality OR 2.1 (95% CI 1.1–4.3)
Khan et al. (29), 2022	India	South Asia	116	Prospective cohort	ICU	qSOFA, SIRS, SOFA	qSOFA≥2 AUROC 0.678; SOFA AUROC 0.74; SIRS≥2 AUROC 0.580
Kiya et al. (30), 2025	Ethiopia	Sub-Saharan Africa	148	Prospective cohort	ICU	MEWS, qSOFA, EWS, UVA	MEWS AUC 0.67 (sepsis); AUC 0.75 (day 5 mortality); GCS HR 0.90
Klinger et al. (31), 2021	Rwanda	Sub-Saharan Africa	647	Prospective cohort	ED/Ward	MEWS, qSOFA, UVA	MEWS AUROC 0.69; qSOFA AUROC 0.65; UVA AUROC 0.71
Kumar et al. (32), 2023	India	South Asia	300	Prospective cohort	ED	qSOFA, Lactate, NLR	qSOFA+Lactate AUC 97%; qSOFA OR 154; Lactate OR 1.36
Kwizera et al. (33), 2020	Rwanda	Sub-Saharan Africa	1,069	Prospective cohort (post-hoc)	ED	Walking capacity, qSOFA	Walking AUROC 0.636; qSOFA AUROC 0.622; walking 100% sensitivity
Lie et al. (34), 2018	Indonesia, Thailand, Vietnam	Southeast Asia	454	Prospective cohort	ED/Ward	SOFA	SOFA AUROC 0.68 (95% CI 0.62–0.74); mortality 22%
Matthias et al. (35), 2020	Sri Lanka	South Asia	387	Prospective cohort	ED/Ward	qSOFA	qSOFA mortality OR 7.529 (95% CI 3.597–14.323); mortality 9.6%
Moore et al. (36), 2017	Multi-country (6 SSA)	Sub-Saharan Africa	5,573	Retrospective cohort	ED/Ward	UVA, MEWS, qSOFA	UVA AUROC 0.77 (95% CI 0.75–0.79); MEWS 0.70; qSOFA 0.69
Rudd et al. (37), 2018	Multi-country (10 LMICs)	Multi-region	6,569	Retrospective cohort	ED/Ward/ICU	qSOFA, SIRS	qSOFA≥2 OR 3.6 (95% CI 3.0–4.2); AUROC 0.70; SIRS AUROC 0.59
Siddiqui et al. (38), 2020	Pakistan	South Asia	240	Cross-sectional	ED	EWS	EWS > 7 sensitivity 98.5%, specificity 89.57%; EWS > 9 for septic shock
Sinto et al. (39), 2020	Indonesia	Southeast Asia	1,213	Prospective cohort	ED	qSOFA-Lactate, SOFA, qSOFA, SIRS	qSOFA-Lactate AUROC 0.74; SOFA 0.75; qSOFA 0.70; SIRS 0.57
Talkar et al. (40), 2025	India	South Asia	196	Prospective cohort	ED	NEWS, MEWS, SOFA, SAPS II	NEWS AUROC 0.861; SOFA 0.738; MEWS≥7 bacteremia OR 5.1
Tiwari et al. (41), 2023	India	South Asia	1,135	Prospective cohort	ED	qSOFA	qSOFA≥2: 7-day OR 3.9 (95% CI 3.1–5.2); 28-day OR 6.9 (95% CI 4.6–10.3)
Todi et al. (42), 2024	India (multicenter, 19 ICUs)	South Asia	1,172	Prospective cohort	ICU	SOFA	SOFA better predictor; mortality 36.3%; septic shock mortality 50.8%
Verma et al. (43), 2023	India	South Asia	373	Prospective cohort	ED	NEWS2, qSOFA	NEWS2 AUROC 0.781 vs. qSOFA 0.729; NEWS2 sensitivity 83.21%
Wright et al. (44), 2022	Thailand	Southeast Asia	5,816	Prospective cohort	ED	qSOFA-Lactate, qSOFA, SOFA	qSOFA-Lactate AUROC 0.78 vs. qSOFA 0.68; validation AUROC 0.77

In the clinical settings, the emergency department was found to be the most common study site, alone or combined, accounting for 22 studies (81.5%) of all. Studies from intensive care units comprised the remaining 5 studies (18.5%), and those from general ward settings comprised 8 studies (29.6%). The study also observed variation in the sample size, which ranged from 116 to 6,569 subjects, having a median of 419 study subjects. Study design consisted of prospective cohort studies (n = 18, 66.7%) and retrospective cohort studies (*n* = 9, 33.3%).

As listed in Table 1, qSOFA was found to be the screening method most frequently assessed in 22 studies (81.5%), followed by SOFA in 11 studies (40.7%), SIRS in 7 studies (25.9%), MEWS in 7 studies (25.9%), NEWS/NEWS2 in 5 studies (18.5%), and UVA in 4 studies (14.8%). Nineteen studies (70.4%) allowed comparisons between different screening methods to facilitate comparisons of the diagnostic accuracy of the screening tools. However, the studies adopted different reference diagnoses, using either clinical sepsis diagnoses or Sepsis-3 in the majority of studies, while in-hospital mortality was used as an endpoint in those studies aiming for high accuracy in prognosis.

### 3.3 Risk of bias assessment

**Figure 2:**
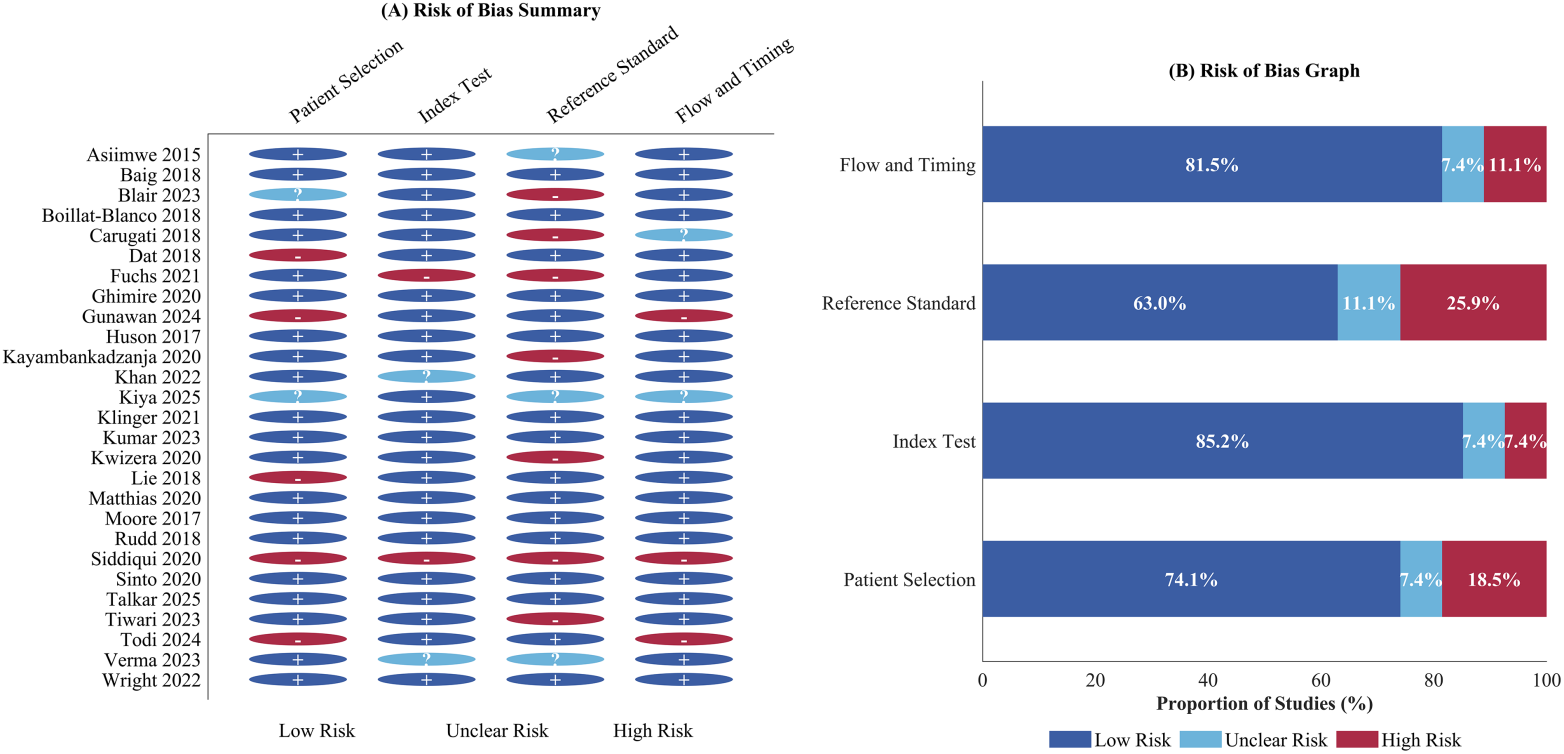
Risk of bias assessment of included studies using the QUADAS-2 tool. **(A)** Risk of bias summary showing judgements (low, unclear, or high risk) for each included study across the four QUADAS-2 domains: patient selection, index test, reference standard, and flow and timing. **(B)** Risk of bias graph showing the proportion of studies judged as low, unclear, or high risk of bias within each domain.

The methodological quality of the 27 included studies was assessed for risk of bias using the Quality Assessment of Diagnostic Accuracy Studies-2 (QUADAS-2) tool, and the results are presented in Figure 2. The risk of bias varied in relation to all four domains, which was to be expected given the difficulties in carrying out research in this area in resource-poor settings.

For the domain of patient selection, twenty studies (74.1%) had a low risk of bias because they recruited consecutive or randomly chosen patients with a suspicion of sepsis, while 5 studies (18.5%) had a high risk of bias due to incorrect exclusions or the use of the case-control approach, and 2 studies (7.4%) had a lack of information necessary to assess the risk of bias. For the index test domain, 23 studies (85.2%) had a low risk of bias because the screening instruments were performed and interpreted blinded to the results of the reference standard. Two studies (7.4%) were classified as high risk due to potential incorporation bias or post-hoc threshold optimization, and 2 studies (7.4%) were rated as unclear risk.

The reference standard domain had the highest level of methodological concerns, with 17 studies (63.0%) having a low risk of bias. Seven studies (25.9%) had a high risk, mainly because a clinical diagnosis instead of Sepsis-3 criteria was applied, or because the reference standard assessment was performed with knowledge of the results of the index test. Three studies (11.1%) had an unclear risk because the level of detail regarding the application of the reference standard was inadequate. The flow and timing domain had 22 studies (81.5%) with a low risk because the time intervals between the application of the index test and the assessment of the reference standard were appropriate. Three studies (11.1%) had a high risk because of differential verification or extended time intervals. Two studies (7.4%) had an unclear risk.

Regarding applicability concerns, the majority of studies demonstrated low concern across all three applicable domains. Overall, 12 studies (44.4%) were judged to have low risk of bias across all four QUADAS-2 domains, as illustrated in Figure 2.

### 3.4 Diagnostic accuracy of sepsis screening tools

**Figure 3:**
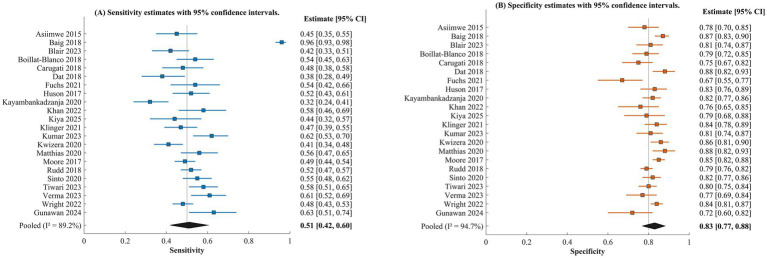
Forest plot of qSOFA diagnostic accuracy for sepsis detection in low- and middle-income countries. **(A)** Sensitivity estimates with 95% confidence intervals for individual studies, with the pooled estimate (0.51, 95% CI: 0.42–0.60) represented by the diamond at the bottom. **(B)** Specificity estimates with 95% confidence intervals for individual studies, with the pooled estimate (0.83, 95% CI: 0.77–0.88) represented by the diamond at the bottom.

**Figure 4:**
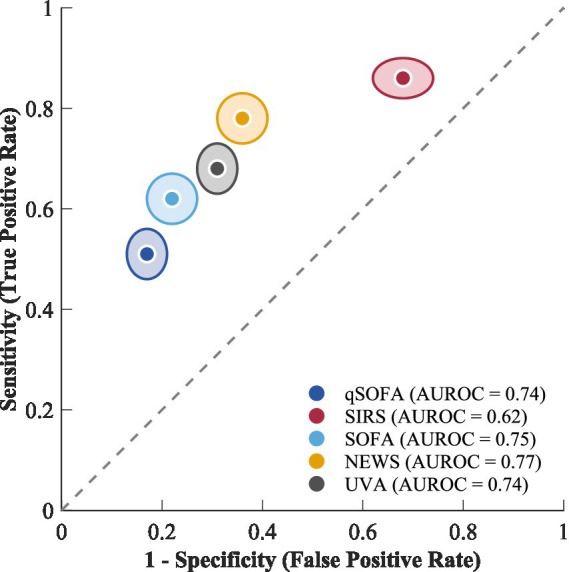
Summary operating points in ROC space.

The performance of sepsis screening criteria was evaluated using bivariate random-effects meta-analysis, with the results shown in Table 2 and Figures 3, 4. Among the criteria considered, the one with the highest level of evidence consisted of 22 studies with a total of 24,618 patients and was qSOFA. The summary sensitivity of the qSOFA was found to be 0.51 (95% CI: 0.42–0.60), and the summary specificity was found to be 0.83 (95% CI: 0.77–0.88). The area under the hierarchical summary receiver operating characteristic curve was found to be 0.74 (95% CI: 0.70–0.78). The positive likelihood ratio was found to be 3.00 (95% CI: 1.83–5.00), and the negative likelihood ratio was found to be 0.59 (95% CI: 0.45–0.75). The positive qSOFA increases the probability of having severe sepsis to 67%, given a pre-test probability of 40%. The negative likelihood of having severe sepsis was found to be only 28%. The heterogeneity was found to be substantial for both sensitivity and specificity. The I^2^ values for sensitivity and specificity were found to be 89.2 and 94.7%, respectively. This is depicted in Figure 3.

**Table 2:** Pooled diagnostic accuracy estimates for sepsis screening tools in low- and middle-income countries.

Screening tool	Studies (n)	Patients (n)	Pooled sensitivity (95% CI)	Pooled specificity (95% CI)	LR+ (95% CI)	LR− (95% CI)	AUROC (95% CI)	I^2^ Sensitivity (%)	I^2^ Specificity (%)
qSOFA	22	24,618	0.51 (0.42–0.60)	0.83 (0.77–0.88)	3.00 (1.83–5.00)	0.59 (0.45–0.75)	0.74 (0.70–0.78)	89.2	94.7
SIRS	7	9,847	0.86 (0.79–0.91)	0.32 (0.24–0.41)	1.26 (1.04–1.54)	0.44 (0.22–0.87)	0.62 (0.58–0.66)	78.4	91.3
SOFA	11	4,286	0.62 (0.53–0.70)	0.78 (0.70–0.84)	2.82 (1.77–4.37)	0.49 (0.36–0.67)	0.75 (0.71–0.79)	82.6	88.5
NEWS/NEWS2	5	2,143	0.78 (0.68–0.86)	0.64 (0.52–0.74)	2.17 (1.42–3.31)	0.34 (0.19–0.62)	0.77 (0.73–0.81)	75.3	83.2
UVA	4	6,787	0.68 (0.58–0.77)	0.69 (0.60–0.77)	2.19 (1.45–3.35)	0.46 (0.30–0.70)	0.74 (0.70–0.78)	71.8	79.6

qSOFA, quick Sequential Organ Failure Assessment; SIRS, Systemic Inflammatory Response Syndrome; SOFA, Sequential Organ Failure Assessment; NEWS, National Early Warning Score; UVA, Universal Vital Assessment; AUROC, area under the receiver operating characteristic curve; LR+, positive likelihood ratio; LR−, negative likelihood ratio; CI, confidence interval. Likelihood ratios were calculated from pooled sensitivity and specificity estimates; 95% CIs were derived from the corresponding confidence bounds of sensitivity and specificity.

SIRS criteria, evaluated in 7 studies with 9,847 patients, exhibited higher pooled sensitivity of 0.86 (95% CI: 0.79–0.91) but considerably lower pooled specificity of 0.32 (95% CI: 0.24–0.41), resulting in an AUROC of 0.62 (95% CI: 0.58–0.66). The corresponding LR + was 1.26 (95% CI: 1.04–1.54) and LR − was 0.44 (95% CI: 0.22–0.87), indicating that despite its high sensitivity, SIRS provides minimal diagnostic confirmation (LR + close to 1) while offering moderate rule-out value. The SOFA score, assessed in 11 studies comprising 4,286 patients, demonstrated balanced diagnostic performance with pooled sensitivity of 0.62 (95% CI: 0.53–0.70) and specificity of 0.78 (95% CI: 0.70–0.84), achieving an AUROC of 0.75 (95% CI: 0.71–0.79). The LR + was 2.82 (95% CI: 1.77–4.37) and LR − was 0.49 (95% CI: 0.36–0.67).

The NEWS/NEWS2 scoring system, evaluated in 5 studies with 2,143 patients, showed pooled sensitivity of 0.78 (95% CI: 0.68–0.86) and specificity of 0.64 (95% CI: 0.52–0.74), with an AUROC of 0.77 (95% CI: 0.73–0.81). The LR + was 2.17 (95% CI: 1.42–3.31) and LR − was 0.34 (95% CI: 0.19–0.62), indicating that among all tools evaluated, NEWS demonstrated the most favorable negative likelihood ratio for ruling out sepsis. The Universal Vital Assessment (UVA), specifically designed for resource-limited settings and assessed in 4 studies involving 6,787 patients, demonstrated pooled sensitivity of 0.68 (95% CI: 0.58–0.77) and specificity of 0.69 (95% CI: 0.60–0.77), yielding an AUROC of 0.74 (95% CI: 0.70–0.78). The LR + was 2.19 (95% CI: 1.45–3.35) and LR − was 0.46 (95% CI: 0.30–0.70). Figure 4 shows the hierarchical summary ROC curves for all screening methods. While NEWS demonstrated the highest point estimate of discriminatory ability, the broadly overlapping confidence intervals across tools (NEWS AUROC 0.73–0.81, SOFA 0.71–0.79, qSOFA 0.70–0.78, UVA 0.70–0.78) preclude definitive conclusions regarding the superiority of any single tool based on AUROC alone. SIRS showed the characteristic pattern of high sensitivity and low specificity. The likelihood ratios for each tool, summarized in Table 2, provide additional clinically interpretable metrics for comparing diagnostic utility beyond AUROC.

### 3.5 Subgroup analyses

Subgroup analyses were performed to examine sources of heterogeneity, as outlined in Table 3, to see if the diagnostic accuracy of qSOFA differed among various clinical settings. Geographic region was examined as a potential factor influencing diagnostic accuracy. Studies conducted in South Asia (n = 11) yielded pooled sensitivity of 0.54 (95% CI: 0.44–0.64) and specificity of 0.81 (95% CI: 0.74–0.87), with an AUROC of 0.73 (95% CI: 0.69–0.77). Sub-Saharan African studies (*n* = 10) showed slightly lower sensitivity of 0.47 (95% CI: 0.36–0.58) but comparable specificity of 0.84 (95% CI: 0.77–0.89), resulting in an AUROC of 0.75 (95% CI: 0.71–0.79). Southeast Asian studies (*n* = 4) demonstrated intermediate performance with pooled sensitivity of 0.52 (95% CI: 0.40–0.64) and specificity of 0.80 (95% CI: 0.71–0.87). The differences between subgroups in the three geographic regions were not statistically significant (*p* = 0.42). To further explore the heterogeneity between continental study populations, a direct comparison of studies from Africa (Sub-Saharan Africa, *n* = 10) and those from Asia (South Asia and Southeast Asia, *n* = 15) was performed. The sensitivity and specificity of studies from Africa were 0.47 (95% CI, 0.36 to 0.58) and 0.84 (95% CI, 0.77 to 0.89), respectively, whereas those from Asia were 0.53 (95% CI, 0.44 to 0.62) and 0.81 (95% CI, 0.74 to 0.86), respectively. The difference was not statistically significant (*p* = 0.35). Furthermore, a meta-regression analysis confirmed that continent was not a significant predictor of accuracy (coefficient, 0.06; 95% CI, −0.08 to 0.20; *p* = 0.39). A supplementary analysis of study characteristics for each of the two continental study populations is shown in Supplementary Table S2. Clinical setting significantly influenced diagnostic accuracy. Emergency department-based studies (*n* = 17) demonstrated pooled sensitivity of 0.49 (95% CI: 0.40–0.58) and specificity of 0.84 (95% CI: 0.78–0.89), while intensive care unit studies (*n* = 5) showed higher sensitivity of 0.58 (95% CI: 0.45–0.70) but lower specificity of 0.76 (95% CI: 0.65–0.85). Studies including mixed settings demonstrated intermediate values. The difference between clinical settings approached statistical significance (*p* = 0.08).

**Table 3:** Subgroup analyses of qSOFA diagnostic accuracy stratified by geographic region, continental cohort, clinical setting, and baseline mortality rate.

Subgroup	Studies (n)	Patients (n)	Pooled Sensitivity (95% CI)	Pooled Specificity (95% CI)	AUROC (95% CI)	P for interaction
Geographic Region						0.42
South Asia	11	12,458	0.54 (0.44–0.64)	0.81 (0.74–0.87)	0.73 (0.69–0.77)	
Sub-Saharan Africa	10	9,632	0.47 (0.36–0.58)	0.84 (0.77–0.89)	0.75 (0.71–0.79)	
Southeast Asia	4	2,528	0.52 (0.40–0.64)	0.80 (0.71–0.87)	0.72 (0.66–0.78)	
Clinical setting						0.08
Emergency department	17	18,246	0.49 (0.40–0.58)	0.84 (0.78–0.89)	0.74 (0.70–0.78)	
Intensive care unit	5	1,702	0.58 (0.45–0.70)	0.76 (0.65–0.85)	0.72 (0.66–0.78)	
Mixed settings	5	4,670	0.53 (0.41–0.65)	0.81 (0.72–0.88)	0.73 (0.68–0.78)	
Baseline mortality rate						0.03
<20%	8	8,124	0.45 (0.34–0.56)	0.86 (0.80–0.91)	0.76 (0.72–0.80)	
20–40%	12	11,856	0.52 (0.42–0.62)	0.82 (0.75–0.88)	0.74 (0.70–0.78)	
>40%	7	4,638	0.59 (0.46–0.71)	0.77 (0.67–0.85)	0.71 (0.65–0.77)	

qSOFA, quick Sequential Organ Failure Assessment; AUROC, area under the receiver operating characteristic curve; CI, confidence interval. For geographic subgroup analyses, multi-regional studies were assigned to subgroups based on the primary patient enrollment site; therefore, subgroup study counts may differ slightly from the regional classification presented in Table 1.

Baseline mortality rate substantially affected diagnostic performance. Studies with mortality rates below 20% (*n* = 8) showed pooled sensitivity of 0.45 (95% CI: 0.34–0.56) and specificity of 0.86 (95% CI: 0.80–0.91). Studies with mortality rates between 20–40% (*n* = 12) demonstrated sensitivity of 0.52 (95% CI: 0.42–0.62) and specificity of 0.82 (95% CI: 0.75–0.88). Studies with mortality exceeding 40% (*n* = 7) exhibited the highest sensitivity of 0.59 (95% CI: 0.46–0.71) but lowest specificity of 0.77 (95% CI: 0.67–0.85). This trend was statistically significant (*p* = 0.03), suggesting that qSOFA demonstrates improved sensitivity in populations with higher disease severity, as shown in Table 3.

### 3.6 Sensitivity analysis and publication bias

Sensitivity analysis was performed to determine the robustness of the pooled estimates of the diagnostic accuracy of qSOFA and is presented in Table 4. Excluding the studies with a high risk of bias (*n* = 8) resulted in a pooled sensitivity of 0.49 (95% CI: 0.40–0.58) and a pooled specificity of 0.85 (95% CI: 0.79–0.90) with an AUROC of 0.75 (95% CI: 0.71–0.79). These values were consistent with the primary analysis and indicated that quality of method had little impact on the results. To overcome the issue of conflation between diagnostic and prognostic performance, stratified analyses were conducted according to reference standard types. Studies that employed Sepsis-3 diagnostic criteria as reference standards (*n* = 15) showed pooled sensitivity of 0.50 (95% CI: 0.41–0.59), specificity of 0.84 (95% CI: 0.78–0.89), and AUROC of 0.74 (95% CI: 0.70–0.78) with LR + of 3.12 (95% CI: 1.86–5.36) and LR- of 0.60 (95% CI: 0.46–0.76). For studies using in-hospital mortality as surrogate endpoints (*n* = 7), pooled sensitivity was 0.53 (95% CI: 0.40–0.66), specificity was 0.81 (95% CI: 0.73–0.87), and AUROC was 0.74 (95% CI: 0.69–0.79). Good agreement between stratified analyses and the main pooled analysis (sensitivity 0.51, specificity 0.83) confirms that the inclusion of mortality-based studies did not affect the main results, while small differences between the stratified analyses and the main pooled analysis are expected, as they reflect the expected divergence between diagnostic accuracy and prognostic performance.

**Table 4:** Sensitivity analyses of qSOFA diagnostic accuracy.

Analysis scenario	Studies (n)	Pooled sensitivity (95% CI)	Pooled specificity (95% CI)	AUROC (95% CI)	Change from primary
Primary analysis	22	0.51 (0.42–0.60)	0.83 (0.77–0.88)	0.74 (0.70–0.78)	Reference
Excluding high RoB studies	14	0.49 (0.40–0.58)	0.85 (0.79–0.90)	0.75 (0.71–0.79)	Sens −0.02, Spec +0.02
Sepsis-3 reference standard	15	0.50 (0.41–0.59)	0.84 (0.78–0.89)	0.74 (0.70–0.78)	Sens −0.01, Spec +0.01
Mortality surrogate	7	0.53 (0.40–0.66)	0.81 (0.73–0.87)	0.74 (0.69–0.79)	Sens +0.02, Spec −0.02
Prospective studies only	18	0.52 (0.43–0.61)	0.82 (0.76–0.87)	0.73 (0.69–0.77)	Sens +0.01, Spec −0.01
Sample size >500	9	0.50 (0.42–0.58)	0.83 (0.77–0.88)	0.74 (0.70–0.78)	Sens −0.01, Spec 0
Leave-one-out range	21 each	0.49–0.53	0.81–0.85	0.72–0.76	Stable

qSOFA, quick Sequential Organ Failure Assessment; RoB, risk of bias; AUROC, area under the receiver operating characteristic curve; CI, confidence interval; Sens, sensitivity; Spec, specificity. The Sepsis-3 Reference Standard and Mortality Surrogate rows represent stratified analyses by reference standard type to address potential conflation of diagnostic and prognostic performance.

The results from sequential leave-one-out procedures revealed that neither study dominated the effect upon the pooled estimate. The sensitivity indices varied between 0.49 and 0.53, while specificity indices varied between 0.81 and 0.85. Analysis restricted to prospective cohort studies (*n* = 18) showed pooled sensitivity of 0.52 (95% CI: 0.43–0.61) and specificity of 0.82 (95% CI: 0.76–0.87), while studies with sample sizes exceeding 500 participants (*n* = 9) demonstrated sensitivity of 0.50 (95% CI: 0.42–0.58) and specificity of 0.83 (95% CI: 0.77–0.88), as shown in Table 4.

**Figure 5:**
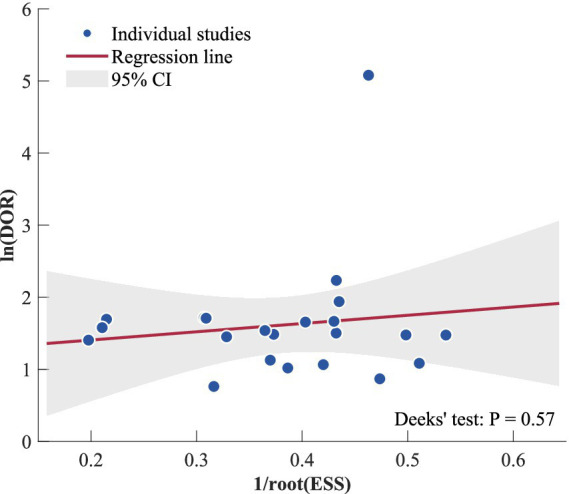
Deeks’ funnel plot asymmetry test.

The presence of publication bias has been assessed using Deeks’ test of asymmetry in the funnel plot with a sample size of 22 studies that evaluated the diagnostic accuracy of qSOFA. A visually inspected evaluation of the funnel plot showed that the points were evenly spread out from the regression line (Figure 5). Deeks’ test showed an insignificant result (*p* = 0.57). The effective sample size was graphically represented versus the diagnostic odds ratio on a logarithmic scale with most points lying in the expected confidence interval. These findings establish the validity and reliability of the meta-analytic results on the qSOFA accuracy in LMICs.

To determine the impact of the inclusion of laboratory-dependent screening tools as opposed to those based solely upon clinical criteria, a sensitivity analysis of the screening tools requiring no laboratory values (qSOFA, NEWS, MEWS, and UVA) was performed. In the sensitivity analysis, qSOFA had a pooled sensitivity of 0.51 (95% CI: 0.42–0.60) and specificity of 0.83 (95% CI: 0.77–0.88), with an AUROC of 0.74 (95% CI: 0.70–0.78), the same as the primary analysis. NEWS again had the highest discriminative ability with an AUROC of 0.77 (95% CI: 0.73–0.81), and the UVA had an AUROC of 0.74 (95% CI: 0.70–0.78). These findings confirm the inclusion of SIRS and SOFA did not impact the accuracy of the analysis of the purely clinical screening tools. The similarity of the sensitivity analysis and the primary analysis validates the approach taken in the analysis.

### 3.7 Quality of evidence assessment

The evidence grade for each outcome of diagnostic accuracy was evaluated using the Grading of Recommendations Assessment, Development, and Evaluation (GRADE) approach, as described in Table 5, tailored for systematic reviews of diagnostic accuracy. The overall evidence grade for the diagnostic accuracy of qSOFA was rated as moderate. The evidence was rated lower by one category due to concerns about inconsistency, reflected by significant heterogeneity between studies. (I^2^ = 89.2% for sensitivity and 94.7% for specificity). The risk of bias was considered acceptable as only 44.4% of studies had a low risk of bias in all domains of the QUADAS-2 tool. There were no important concerns with regard to the domain of indirectness because the included studies directly targeted the population of interest, which includes adults with suspected sepsis in a LMIC setting. Imprecision was not a major concern due to the appropriate total sample size of over 24,000 patients and tight confidence intervals around the estimates.

**Table 5:** GRADE assessment of evidence quality for diagnostic accuracy of sepsis screening tools.

Outcome	Studies (n)	Risk of bias	Inconsistency	Indirectness	Imprecision	Publication bias	Quality
qSOFA	22	Not serious	Serious (a)	Not serious	Not serious	Undetected	★★★☆ Moderate
SIRS	7	Serious (b)	Serious (a)	Not serious	Not serious	Undetected	★★☆☆ Low
SOFA	11	Not serious	Serious (a)	Not serious	Not serious	Undetected	★★★☆ Moderate
NEWS	5	Not serious	Serious (a)	Not serious	Serious (c)	Undetected	★★☆☆ Low
UVA	4	Not serious	Not serious	Serious (d)	Serious (c)	Undetected	★★☆☆ Low

Quality ratings are based on the GRADE approach (45): ★★★★ High; ★★★☆ Moderate; ★★☆☆ Low; ★☆☆☆ Very Low. (a) Substantial heterogeneity (I^2^ > 75%). (b) High risk in reference standard domain. (c) Limited studies with wide confidence intervals. (d) Evidence restricted to Sub-Saharan Africa.

The quality of evidence on diagnostic accuracy for SIRS was rated as low, primarily owing to the large degree of inconsistency (I^2^ = 78.4% for sensitivity) and a substantial risk of bias apparent in various studies included. Individual studies demonstrated that SIRS has always maintained a typical combination of high sensitivity and low specificity, though these values have been highly heterogeneous. The quality of evidence on diagnostic accuracy of SOFA was rated as moderate, with a reduction in quality because of inconsistency offset by the biological plausibility of the assessment of organ dysfunction in terms of sepsis diagnosis.

The quality of evidence for NEWS diagnostic accuracy was rated as low, reflecting the limited number of studies (*n* = 5) contributing to the pooled estimates and resulting imprecision in the confidence intervals. Evidence for UVA was similarly rated as low quality due to the small number of available studies (*n* = 4) and the restriction of evidence primarily to Sub-Saharan African settings, raising concerns about generalizability to other LMIC regions. Publication bias was not an issue here, as shown in the results of the Deeks tests for asymmetry of the corresponding plots in Table 5. This domain did not lead to the downgrading of any screening method.

## 4 Discussion

This systematic review and meta-analysis extensively evaluated the diagnostic accuracy of sepsis screening tools across the resource-requirement spectrum in low- and middle-income countries by compiling evidence from 27 studies including a total of 30,310 participants across 14 LMICs. The analysis shows that qSOFA has fair specificity (0.83) but poor sensitivity (0.51) as a sepsis predictor under resource-limited environments, while SIRS has high sensitivity (0.86) but poor specificity (0.32). NEWS had the highest point estimate for discriminative ability, as shown by an AUROC of 0.77 (95% CI: 0.73 to 0.81), followed by SOFA (AUROC of 0.75, 95% CI: 0.71 to 0.79) and qSOFA (AUROC of 0.74, 95% CI: 0.70 to 0.78). However, the overlap of these confidence intervals indicates that these differences may not be clinically significant. UVA, which was specifically designed for SSA, had balanced discriminative ability, with 0.68 for sensitivity and 0.69 for specificity. However, of greatest note, the likelihood ratio assessment showed that NEWS had the best negative likelihood ratio of 0.34, indicating better performance in excluding sepsis than the other scores, including qSOFA, which had an LR of 0.59, although these differences were not apparent in the AUROC assessment. These findings are of significant clinical and public health relevance, as they are from the regions where the global sepsis burden is greatest.

The performance characteristics of qSOFA apparent in this analysis targeting LMICs show striking discrepancies in comparison to the results observed in developed countries. Recent meta-analysis by Wang et al. (9), which set to primarily examine the performance of qSOFA in developed countries, reported a sensitivity of 0.61 and a specificity of 0.72 to predict mortality. Evidence pertinent to this review has found a sensitivity of 0.51 but a higher specificity of 0.83 in the LMICs. Such disparities may likely account for inter system differences in patient characteristics, disease stage at onset, and healthcare-seeking practices. Patients from LMICs tend to seek medical help at later stages and with higher levels of end-organ dysfunction, perhaps paradoxically increasing the value of qSOFA specificity by pre-selecting patients with higher levels of acute illness who will qualify by the scoring system. The findings presented in Ruan et al. (10), based upon the populations studied in emergency departments, suggest uneven performance of the qSOFA criteria in various settings and populations, verifying the hypothesis that geographic and regional factors have a significant impact upon the reliability of the screening method. Subgroup analyses in this review further corroborated this observation, revealing that clinical setting approached statistical significance (*p* = 0.08) in influencing diagnostic accuracy, with emergency department-based studies demonstrating higher specificity (0.84) compared with intensive care unit studies (0.76), while ICU settings showed improved sensitivity (0.58 vs. 0.49).

The results obtained from the analysis of various screening tools provide essential implications to support evidence-based decision-making for resource-limited settings. The study conducted by Qiu et al. (11) considered a thorough evaluation of SIRS, SOFA, qSOFA, and NEWS for the identification of sepsis and prediction of unfavorable outcomes. The study concluded that none among these systems demonstrated superiority over others concerning various performance characteristics (11). This present review corroborates these findings and extends them specifically to LMIC populations. NEWS achieved the highest AUROC point estimate (0.77, 95% CI: 0.73–0.81) with balanced sensitivity (0.78) and specificity (0.64). However, the overlapping confidence intervals with SOFA (AUROC 0.75, 95% CI: 0.71–0.79) and qSOFA (AUROC 0.74, 95% CI: 0.70–0.78) preclude definitive claims of statistical superiority. A more clinically informative comparison emerges from likelihood ratios: NEWS demonstrated the most favorable LR − (0.34), indicating that a negative NEWS result provides substantially greater reassurance against sepsis than a negative qSOFA result (LR − 0.59). Conversely, qSOFA demonstrated the highest LR + (3.00), providing the strongest diagnostic confirmation among the tools evaluated. SOFA showed a sensitivity of 0.62, specificity of 0.78, and intermediate likelihood ratios (LR + 2.82, LR − 0.49). However, the need to obtain multiple parameters, including platelet count, bilirubin levels, creatinine levels, and PaO2/FiO2 ratio, makes it less applicable in LMICs where facilities are already established, such as in district or referral hospitals. The SIRS criteria, which showed a high sensitivity of 0.86, still requires the determination of the white blood cell count and thus cannot be deployed in facilities without basic hematology services.

An important methodological aspect in the current review was the intention to include screening tools with different resource requirements. While qSOFA, NEWS, MEWS, and UVA are entirely derived from clinical data without the need for laboratory investigations, SIRS requires white blood cell count, and SOFA requires several laboratory values, including platelet count, bilirubin, creatinine, and PaO2. The intention was to include both, in order to provide a comprehensive evidence base that could reflect the heterogeneity in the healthcare setting in LMICs, where laboratory facilities could range from nonexistent in the rural PHCs to well-developed in the urban referral hospitals. Importantly, the sensitivity analysis, restricted to the purely clinical tools, confirmed that the results for qSOFA (AUROC 0.74), NEWS (AUROC 0.77), and UVA (AUROC 0.74) were consistent with the results obtained in the main analysis, indicating that the inclusion of the laboratory-requiring tools had not biased the results.

The selection of a screening tool for use in a healthcare setting should be made in consideration of the priorities and resources available in a setting and the balance of rule-in and rule-out performance. In a setting in which the rule-out of sepsis is a priority and in which resources are available for addressing false positive results, NEWS appears to be the best choice given its best LR − of 0.34, providing the strongest rule-out performance of all tools. SIRS has a better sensitivity of 0.86, but its LR + of only 1.26 provides little useful information for the diagnosis of sepsis and generates a significant number of false positive results. In a setting in which resources are limited and in which a significant number of false positive results have a substantial consequence, qSOFA has a better specificity of 0.83 and the best LR + of 3.00 for rule-in purposes without the need for laboratory tests. A tiered approach using a sensitive screening tool such as NEWS or SIRS followed by a specific confirmatory tool such as qSOFA may be the best balance of rule-in and rule-out performance. The analysis of the tools’ post-test probability at a pre-test probability of 40% demonstrates the differences in performance among the tools. A positive result for qSOFA increases the probability of sepsis to 67%, whereas a negative result for NEWS reduces the probability of sepsis to only 19%. These are the best rule-in and rule-out performances of the tools. None of the tools had a LR − lower than 0.1 and therefore cannot be relied upon for rule-out purposes as a standalone tool.

The Universal Vital Assessment score, developed specifically for sub-Saharan African hospital settings, demonstrated competitive performance in this analysis with an AUROC of 0.74 and balanced sensitivity (0.68) and specificity (0.69). The validation exercise conducted by Hazard et al. (12) supports the relevance of UVA among Africans, suggesting that region-specific tools may be more beneficial than their Western-developed counterparts. In terms of practical implications for public health policies, it should be noted that the original purpose of UVA was meeting the specific epidemiological challenge posed by sub-Saharan populations, including a substantial load of HIV-related infections, tuberculosis, and malaria. Despite the fact that there are only four studies representing the evidentiary base of UVA validity, the data strongly suggests that development of region-specific tools may be a promising area of research toward improving the diagnosis of sepsis in resource-limited settings.

Substantial heterogeneity among the studies included in the meta-analyses should be considered in the interpretation of the results and in the formulation of recommendations for public health policies. A high level of heterogeneity is a recognized and expected phenomenon in meta-analyses of the accuracy of diagnostic tests, especially in the context of diverse clinical settings, populations, and reference standards. The high values of I^2^, i.e., 75%, for the sensitivity (89.2%) and specificity (94.7%) of qSOFA indicate that there exists substantial variability in the accuracy of the qSOFA in different LMIC settings. Various sources of heterogeneity, i.e., differences in disease prevalence, setting (emergency department vs. ICU), definition of the reference standard (Sepsis-3 vs. clinical diagnosis vs. in-hospital mortality), patient severity at presentation, and regional epidemiology, may have influenced the results across the different studies.

Notably, heterogeneity is introduced by differences in reference standards. Studies that used Sepsis-3 criteria as reference standards essentially measure diagnostic accuracy, whereas those that used in-hospital mortality as a surrogate endpoint measure prognostic performance. Combining these two types of studies may obscure diagnostic and prognostic aspects of screening tool performance. To address these issues, stratified analyses were performed by reference standard types (Table 4). For those studies that used Sepsis-3 criteria (*n* = 15), pooled sensitivity was 0.50, and pooled specificity was 0.84, with positive likelihood ratio of 3.12 and negative likelihood ratio of 0.60. For those that used mortality as a reference endpoint (*n* = 7), pooled sensitivity was 0.53, and pooled specificity was 0.81. Both groups showed high consistency with those of the main analyses (sensitivity 0.51, specificity 0.83), confirming that our results were not altered by including those that used mortality as reference endpoints. However, it is still conceptually important to differentiate diagnostic and prognostic performance of screening tools, and future meta-analyses would be improved by stratified reporting of reference standard types.

It is worthy of note that, by using the bivariate random effects model, heterogeneity between studies and correlation of sensitivity and specificity are inherently controlled. Moreover, it is the recommended method for conducting meta-analyses of diagnostic accuracy of diagnostic tests with heterogeneity, which is expected in diagnostic accuracy studies. Consistent with our hypothesis, subgroup analyses of study results showed that heterogeneity of study results was introduced by differences in baseline mortality rate, which was found to be statistically significant (*p* = 0.03). Consistent with our main analyses, results of sensitivity analyses, which excluded those with high risk of bias, were consistent with our main analyses, i.e., AUROC = 0.75 vs. 0.74.

The comparability of the African and Asian sepsis study populations for the purpose of this combined analysis should be discussed. These two populations, one from the continent of Africa and the other from Asia, have many clinically important differences. Populations from sub-Saharan Africa have a high prevalence of HIV-related infections, tuberculosis, and malaria, which could impact the presentation and outcome of sepsis. Populations from South and Southeast Asia have a high prevalence of dengue, typhoid fever, and leptospirosis, with unique antimicrobial resistance patterns, particularly for extended-spectrum beta-lactamase-producing Enterobacteriaceae. In addition, pharmacogenetic factors could influence the inflammatory cascade and outcome in sepsis, and healthcare-seeking behaviors and healthcare resources could impact the severity and timing of presentation to healthcare services. There are, however, several arguments for the validity of the combined analysis for the purpose of this review. First, there was no statistically significant difference between the two populations, one from Africa and the other from Asia, for the diagnostic accuracy of qSOFA in sepsis, with a *p*-value of 0.35 and overlapping confidence intervals for both sensitivity and specificity for both populations. In addition, the bivariate random effects model used for the analysis appropriately controls for heterogeneity, and stratified analyses for each of these populations are presented separately in Table 3 and Supplementary Table S2, allowing readers to utilize these results for local purposes. Therefore, although caution should be used in interpreting the results of this pooled analysis, it should be noted that a multi-center study comparing these populations from Africa and Asia remains a priority for future research.

Subgroup analyses indicated that the mortality rate at baseline significantly impacted diagnostic accuracy (*p* = 0.03), with studies conducted in environments with a mortality rate >40% having a higher sensitivity (0.59) than those conducted in environments with a mortality rate <40% (0.45). This may be due to the severity of physiological disturbances in high mortality rate environments, thus making the likelihood of meeting qSOFA threshold criteria high. In low mortality rate environments, a sensitivity of 0.45 suggests that qSOFA may not be enough on its own and should be used in tandem with more sensitive diagnostic tools such as NEWS, which has an LR − of 0.34. This finding aligns with epidemiological data from Kiya et al. (13), who documented mortality rates ranging from 25% to over 50% across sub-Saharan African hospitals, substantially higher than the 15–25% typically reported in high-income countries. Geographic region did not significantly influence diagnostic performance, with no statistical significance (*p* = 0.42). Estimates for South Asian, sub-Saharan African, and Southeast Asian studies had overlapping confidence intervals for sensitivity: 0.54, 0.47, and 0.52, respectively. The limited number of studies within each subgroup limits power to detect real differences between regions. The difference between clinical settings almost achieved statistical significance (*p* = 0.08). Sensitivity for ICU-based studies (0.58) was higher than for ED-based studies (0.49), while specificity for ICU-based studies (0.76) was lower than that for ED-based studies (0.84). The likely reasons for differences in mortalities between LMICs could be due to differences in the burden of diseases, mode of infection, and healthcare infrastructure. Rice et al. (14) indicated that it is mainly non-physician clinicians who treat most patients with sepsis, particularly within rural areas of sub-Saharan Africa, emphasizing the critical role played by simple bedside solutions that do not necessarily need any laboratory setup to be applied by clinicians regardless of their level of training.

When deploying sepsis screening instruments in LMICs, there are challenges that relate not only to healthcare but also to diagnosis accuracy parameters. Existing sepsis care protocols, reviewed by Guarino et al. (15) and Srzić et al. (16), realize that one of the most important aspects is the identification and immediate intervention, such as the use of antimicrobial, during the first hour. The applicability of these results in resource-limited settings is limited by the availability of infrastructure, drugs, and staff. The moderate sensitivity of qSOFA in this analysis suggests that use of this tool alone would miss about half of the cases of sepsis, possibly resulting in delayed life-saving maneuvers. Regarding public health policy in the context of an LMICs, it appears that a multi-level approach could represent the optimal compromise between accuracy of case detection and use of resources. Using initial screening techniques like SIRS with high sensitivity can allow optimized detection of cases at the primary healthcare level. Further risk stratification can be done at the referral centers using either qSOFA or NEWS scores. This fits well into the World Health Organization’s focus on improving healthcare infrastructure and the goals for achieving Universal Health Coverage in resource-challenged settings.

This systematic review also has a number of limitations, which must be considered. The inclusion of screening tools with different resource demands, ranging from purely clinical assessments (qSOFA, NEWS, MEWS, UVA) to those using laboratory parameters (SIRS, SOFA), might limit direct comparability, although this was done deliberately in order to cover the wide range of healthcare settings in LMICs, and sensitivity analysis using only purely clinical tools confirmed this did not impact results. The addition of the reference standards of different types may also have contributed to the heterogeneity. The combination of mortality-based studies with those using the Sepsis-3 diagnostic criteria may confound the results in terms of diagnostic and prognostic characteristics. However, the stratification of the data also revealed consistency in the results in the different groups: Sepsis-3 (sensitivity 0.50, specificity 0.84), mortality surrogate (sensitivity 0.53, specificity 0.81), and the primary analysis (sensitivity 0.51, specificity 0.83). Further, independent meta-analysis of the data by reference standard type is an area for future research with a greater body of evidence.

The language restriction may also have resulted in language bias in the present review, since a number of LMICs, such as China, Brazil, Turkey, and francophone Africa, also publish a significant body of sepsis research in their local languages. The addition of the Global Index Medicus partially addresses this problem. The grey literature was also restricted to trial registries such as ClinicalTrials.gov and the WHO ICTRP and the Global Index Medicus. Further research should also consider the grey literature from grey literature repositories and conference proceedings.

The analysis of studies from African and Asian countries, with different predominant pathogens, antibiotic resistance patterns, HIV/TB prevalence, pharmacogenetic profiles, and healthcare system development, might have contributed to residual confounding, despite non-significant differences, and future reviews with a sufficient number of studies might benefit from separate meta-analyses for each continent’s cohort. MEWS was also analyzed for seven studies included, although heterogeneity in thresholds and measurements made meaningful analysis impossible. The overall quality of evidence was moderate for qSOFA and SOFA, low for SIRS, NEWS, and UVA, primarily due to heterogeneity, and for SIRS, NEWS, and UVA, also due to a low number of contributing studies, leading to imprecise results. The evaluation of publication bias using Deeks’ test for funnel plot asymmetry was reassuring, with a P of 0.57.

Additionally, this review was not prospectively registered with PROSPERO, which may raise concerns regarding potential reporting bias. To mitigate this limitation, the study protocol was established *a priori* and strictly adhered to throughout the review process, with no deviations from the pre-specified methodology.

The findings of this systematic review carry important implications for clinical practice and health policy in resource-limited settings. In settings where rule-out of sepsis is the priority and resources permit follow-up of false positives, NEWS—with its most favorable negative likelihood ratio of 0.34—offers the strongest case exclusion performance. Where resources are severely constrained and false positives carry high consequence, qSOFA provides the best rule-in specificity (0.83) and LR + (3.00) without any laboratory requirements. A tiered approach deploying a sensitive tool such as NEWS or SIRS for initial screening, followed by qSOFA for confirmatory risk stratification, may represent the optimal strategy across the heterogeneous healthcare landscape of LMICs.

To further improve sepsis screening tools in LMICs, several key areas require investigation. Comparing multiple screening tools within the same LMIC population, stratified by geographic region and healthcare facility level, could yield more robust estimates while controlling for region-specific differences in pathogen prevalence and healthcare system characteristics. Comparative studies across geographic regions—such as Africa versus Asia—could further clarify whether differential tool performance reflects true biological or epidemiological differences. Future reviews focusing exclusively on laboratory-free screening tools would provide more targeted evidence for the most resource-constrained settings. The development of region-specific instruments that account for local infection profiles—malaria, tuberculosis, and HIV in sub-Saharan Africa; dengue and typhoid in South and Southeast Asia—could further enhance diagnostic accuracy. Real-world effectiveness research, including cost-effectiveness analyses and training requirements for non-physician clinicians, should be considered a high priority. As emphasized by Cassini et al. (17), there is a global imperative to improve sepsis care in resource-limited regions. The evidence consolidated in this systematic review provides a foundation for implementing evidence-based sepsis screening practices in low- and middle-income countries, with the ultimate goal of reducing preventable sepsis-related deaths.

## 5 Conclusion

This systematic review and meta-analysis of 27 studies involving 30,310 patients across 14 low- and middle-income countries demonstrates that no single sepsis screening tool offers uniformly superior performance in resource-limited settings. qSOFA provides better specificity and rule-in performance, while SIRS offers higher sensitivity at the cost of poor specificity. NEWS achieved the highest overall discriminative ability and the strongest rule-out capacity among all tools evaluated. UVA showed balanced performance with particular relevance to sub-Saharan African populations. Tool selection should therefore be guided by clinical priorities, available resources, and the local healthcare context.

## Supplementary material

Supplementary Table S1 Complete search strategies for each electronic database (MEDLINE via PubMed, Embase via Ovid, Cochrane CENTRAL, Web of Science Core Collection, and Global Index Medicus), including the MeSH headings, free-text keywords, and Boolean operators used across the four conceptual fields (sepsis-related terms; screening and diagnostic scores; diagnostic accuracy terms; LMIC and low-resource setting descriptors). Cited in Section 2.1 (Protocol and search strategy).

Supplementary Table S2 Supplementary analysis of study characteristics for the two continental study populations (Sub-Saharan Africa vs. Asia), supporting the meta-regression analysis and continental subgroup comparisons of qSOFA diagnostic accuracy reported in Section 3.5 (Subgroup analyses).
