# Supplementary material for: Sepsis screening tools in resource-limited settings: a systematic review and meta-analysis of diagnostic accuracy in low- and middle-income countries
Source: Front Public Health. 2026 May 20;14:1782420. doi: 10.3389/fpubh.2026.1782420 (PMC13230155; doi:10.3389/fpubh.2026.1782420)
Supplement: Supplementary Table S1 — Complete search strategies for each electronic database (MEDLINE via PubMed, Embase via Ovid, Cochrane CENTRAL, Web of Science Core Collection, and Global Index Medicus), including the MeSH headings, free-text keywords, and Boolean operators used across the four conceptual fields (sepsis-related terms; screening and diagnostic scores; diagnostic accuracy terms; LMIC and low-resource setting descriptors). Cited in Section 2.1 (Protocol and search strategy). [file Table_1.DOCX]

**Supplementary Table S1. Complete Search Strategies for All Databases**

| **Database** | **Search Block** | **Search Terms** |
| --- | --- | --- |
| PubMed/MEDLINE | #1 Sepsis | "Sepsis"[MeSH Terms] OR "Sepsis"[Title/Abstract] OR "severe sepsis"[Title/Abstract] OR "septic shock"[Title/Abstract] OR "Septicemia"[Title/Abstract] OR "Septicaemia"[Title/Abstract] |
|  | #2 Screening Tools | "qSOFA"[Title/Abstract] OR "quick SOFA"[Title/Abstract] OR "quick sequential organ failure assessment"[Title/Abstract] OR "SIRS"[Title/Abstract] OR "systemic inflammatory response syndrome"[Title/Abstract] OR "NEWS"[Title/Abstract] OR "NEWS2"[Title/Abstract] OR "national early warning score"[Title/Abstract] OR "MEWS"[Title/Abstract] OR "modified early warning score"[Title/Abstract] OR "SOFA"[Title/Abstract] OR "sequential organ failure assessment"[Title/Abstract] OR "UVA"[Title/Abstract] OR "universal vital assessment"[Title/Abstract] OR "early warning score*"[Title/Abstract] OR "sepsis screening"[Title/Abstract] OR "bedside screening"[Title/Abstract] |
|  | #3 Diagnostic Accuracy | "Sensitivity and Specificity"[MeSH Terms] OR "Sensitivity"[Title/Abstract] OR "Specificity"[Title/Abstract] OR "diagnostic accuracy"[Title/Abstract] OR "ROC Curve"[MeSH Terms] OR "ROC"[Title/Abstract] OR "AUC"[Title/Abstract] OR "AUROC"[Title/Abstract] OR "area under the curve"[Title/Abstract] OR "receiver operating characteristic"[Title/Abstract] OR "Predictive Value of Tests"[MeSH Terms] OR "predictive value"[Title/Abstract] OR "likelihood ratio"[Title/Abstract] OR "diagnostic performance"[Title/Abstract] OR "validation"[Title/Abstract] |
|  | #4 LMICs | "Developing Countries"[MeSH Terms] OR "LMIC"[Title/Abstract] OR "LMICs"[Title/Abstract] OR "low income"[Title/Abstract] OR "middle income"[Title/Abstract] OR "resource limited"[Title/Abstract] OR "resource-limited"[Title/Abstract] OR "resource poor"[Title/Abstract] OR "low-resource"[Title/Abstract] OR "Sub-Saharan Africa"[Title/Abstract] OR "South Asia"[Title/Abstract] OR "Southeast Asia"[Title/Abstract] OR "India"[Title/Abstract] OR "Pakistan"[Title/Abstract] OR "Nepal"[Title/Abstract] OR "Sri Lanka"[Title/Abstract] OR "Indonesia"[Title/Abstract] OR "Vietnam"[Title/Abstract] OR "Thailand"[Title/Abstract] OR "Cambodia"[Title/Abstract] OR "Uganda"[Title/Abstract] OR "Rwanda"[Title/Abstract] OR "Malawi"[Title/Abstract] OR "Tanzania"[Title/Abstract] OR "Ethiopia"[Title/Abstract] OR "Ghana"[Title/Abstract] |
|  | #5 Population | "Adult"[MeSH Terms] OR "adult*"[Title/Abstract] OR "Aged"[MeSH Terms] |
|  | #6 Combined | #1 AND #2 AND #3 AND #4 AND #5 |
| Embase | #1 Sepsis | 'sepsis'/exp OR 'sepsis':ti,ab OR 'severe sepsis':ti,ab OR 'septic shock':ti,ab OR 'septicemia':ti,ab OR 'septicaemia':ti,ab |
|  | #2 Screening Tools | 'qSOFA':ti,ab OR 'quick SOFA':ti,ab OR 'quick sequential organ failure assessment':ti,ab OR 'SIRS':ti,ab OR 'systemic inflammatory response syndrome':ti,ab OR 'NEWS':ti,ab OR 'NEWS2':ti,ab OR 'national early warning score':ti,ab OR 'MEWS':ti,ab OR 'modified early warning score':ti,ab OR 'SOFA':ti,ab OR 'sequential organ failure assessment':ti,ab OR 'UVA':ti,ab OR 'universal vital assessment':ti,ab OR 'early warning score*':ti,ab OR 'sepsis screening':ti,ab OR 'bedside screening':ti,ab |
|  | #3 Diagnostic Accuracy | 'sensitivity and specificity'/exp OR 'sensitivity':ti,ab OR 'specificity':ti,ab OR 'diagnostic accuracy':ti,ab OR 'receiver operating characteristic'/exp OR 'ROC':ti,ab OR 'AUC':ti,ab OR 'AUROC':ti,ab OR 'area under the curve':ti,ab OR 'receiver operating characteristic':ti,ab OR 'predictive value':ti,ab OR 'likelihood ratio':ti,ab OR 'diagnostic performance':ti,ab OR 'validation':ti,ab |
|  | #4 LMICs | 'developing country'/exp OR 'LMIC':ti,ab OR 'LMICs':ti,ab OR 'low income':ti,ab OR 'middle income':ti,ab OR 'resource limited':ti,ab OR 'resource-limited':ti,ab OR 'resource poor':ti,ab OR 'low-resource':ti,ab OR 'Sub-Saharan Africa':ti,ab OR 'South Asia':ti,ab OR 'Southeast Asia':ti,ab OR 'India':ti,ab OR 'Pakistan':ti,ab OR 'Nepal':ti,ab OR 'Sri Lanka':ti,ab OR 'Indonesia':ti,ab OR 'Vietnam':ti,ab OR 'Thailand':ti,ab OR 'Cambodia':ti,ab OR 'Uganda':ti,ab OR 'Rwanda':ti,ab OR 'Malawi':ti,ab OR 'Tanzania':ti,ab OR 'Ethiopia':ti,ab OR 'Ghana':ti,ab |
|  | #5 Population | 'adult'/exp OR 'adult*':ti,ab OR 'aged'/exp |
|  | #6 Combined | #1 AND #2 AND #3 AND #4 AND #5 |
| Cochrane CENTRAL | #1 Sepsis | [mh "Sepsis"] OR "sepsis":ti,ab,kw OR "severe sepsis":ti,ab,kw OR "septic shock":ti,ab,kw OR "septicemia":ti,ab,kw OR "septicaemia":ti,ab,kw |
|  | #2 Screening Tools | "qSOFA":ti,ab,kw OR "quick SOFA":ti,ab,kw OR "quick sequential organ failure assessment":ti,ab,kw OR "SIRS":ti,ab,kw OR "systemic inflammatory response syndrome":ti,ab,kw OR "NEWS":ti,ab,kw OR "NEWS2":ti,ab,kw OR "national early warning score":ti,ab,kw OR "MEWS":ti,ab,kw OR "modified early warning score":ti,ab,kw OR "SOFA":ti,ab,kw OR "sequential organ failure assessment":ti,ab,kw OR "UVA":ti,ab,kw OR "universal vital assessment":ti,ab,kw OR "early warning score*":ti,ab,kw OR "sepsis screening":ti,ab,kw OR "bedside screening":ti,ab,kw |
|  | #3 Diagnostic Accuracy | [mh "Sensitivity and Specificity"] OR "sensitivity":ti,ab,kw OR "specificity":ti,ab,kw OR "diagnostic accuracy":ti,ab,kw OR [mh "ROC Curve"] OR "ROC":ti,ab,kw OR "AUC":ti,ab,kw OR "AUROC":ti,ab,kw OR "area under the curve":ti,ab,kw OR "receiver operating characteristic":ti,ab,kw OR "predictive value":ti,ab,kw OR "likelihood ratio":ti,ab,kw OR "diagnostic performance":ti,ab,kw OR "validation":ti,ab,kw |
|  | #4 LMICs | [mh "Developing Countries"] OR "LMIC":ti,ab,kw OR "LMICs":ti,ab,kw OR "low income":ti,ab,kw OR "middle income":ti,ab,kw OR "resource limited":ti,ab,kw OR "resource-limited":ti,ab,kw OR "resource poor":ti,ab,kw OR "low-resource":ti,ab,kw OR "Sub-Saharan Africa":ti,ab,kw OR "South Asia":ti,ab,kw OR "Southeast Asia":ti,ab,kw OR "India":ti,ab,kw OR "Pakistan":ti,ab,kw OR "Nepal":ti,ab,kw OR "Sri Lanka":ti,ab,kw OR "Indonesia":ti,ab,kw OR "Vietnam":ti,ab,kw OR "Thailand":ti,ab,kw OR "Cambodia":ti,ab,kw OR "Uganda":ti,ab,kw OR "Rwanda":ti,ab,kw OR "Malawi":ti,ab,kw OR "Tanzania":ti,ab,kw OR "Ethiopia":ti,ab,kw OR "Ghana":ti,ab,kw |
|  | #5 Population | [mh "Adult"] OR "adult*":ti,ab,kw OR [mh "Aged"] |
|  | #6 Combined | #1 AND #2 AND #3 AND #4 AND #5 |
| Web of Science | #1 Sepsis | TS=(sepsis OR "severe sepsis" OR "septic shock" OR septicemia OR septicaemia) |
|  | #2 Screening Tools | TS=(qSOFA OR "quick SOFA" OR "quick sequential organ failure assessment" OR SIRS OR "systemic inflammatory response syndrome" OR NEWS OR NEWS2 OR "national early warning score" OR MEWS OR "modified early warning score" OR SOFA OR "sequential organ failure assessment" OR UVA OR "universal vital assessment" OR "early warning score*" OR "sepsis screening" OR "bedside screening") |
|  | #3 Diagnostic Accuracy | TS=(sensitivity OR specificity OR "diagnostic accuracy" OR ROC OR AUC OR AUROC OR "area under the curve" OR "receiver operating characteristic" OR "predictive value" OR "likelihood ratio" OR "diagnostic performance" OR validation) |
|  | #4 LMICs | TS=(LMIC OR LMICs OR "low income" OR "middle income" OR "resource limited" OR "resource-limited" OR "resource poor" OR "low-resource" OR "developing countr*" OR "Sub-Saharan Africa" OR "South Asia" OR "Southeast Asia" OR India OR Pakistan OR Nepal OR "Sri Lanka" OR Indonesia OR Vietnam OR Thailand OR Cambodia OR Uganda OR Rwanda OR Malawi OR Tanzania OR Ethiopia OR Ghana) |
|  | #5 Population | TS=(adult* OR aged) |
|  | #6 Combined | #1 AND #2 AND #3 AND #4 AND #5 |
| Global Index Medicus | #1 Sepsis | (tw:(sepsis)) OR (tw:("severe sepsis")) OR (tw:("septic shock")) OR (tw:(septicemia)) OR (tw:(septicaemia)) |
|  | #2 Screening Tools | (tw:(qSOFA)) OR (tw:("quick SOFA")) OR (tw:(SIRS)) OR (tw:("systemic inflammatory response syndrome")) OR (tw:(NEWS)) OR (tw:(NEWS2)) OR (tw:(MEWS)) OR (tw:(SOFA)) OR (tw:("sequential organ failure assessment")) OR (tw:(UVA)) OR (tw:("universal vital assessment")) OR (tw:("early warning score")) OR (tw:("sepsis screening")) OR (tw:("bedside screening")) |
|  | #3 Diagnostic Accuracy | (tw:(sensitivity)) OR (tw:(specificity)) OR (tw:("diagnostic accuracy")) OR (tw:(ROC)) OR (tw:(AUC)) OR (tw:(AUROC)) OR (tw:("area under the curve")) OR (tw:("receiver operating characteristic")) OR (tw:("predictive value")) OR (tw:("likelihood ratio")) OR (tw:("diagnostic performance")) OR (tw:(validation)) |
|  | #4 LMICs | (tw:(LMIC)) OR (tw:(LMICs)) OR (tw:("low income")) OR (tw:("middle income")) OR (tw:("resource limited")) OR (tw:("resource poor")) OR (tw:("low-resource")) OR (tw:("Sub-Saharan Africa")) OR (tw:("South Asia")) OR (tw:("Southeast Asia")) |
|  | #5 Population | (tw:(adult)) OR (tw:(aged)) |
|  | #6 Combined | #1 AND #2 AND #3 AND #4 AND #5 |

Abbreviations: MeSH, Medical Subject Headings; ti,ab, title and abstract; kw, keyword; TS, topic search; tw, text word; exp, exploded term; qSOFA, quick Sequential Organ Failure Assessment; SIRS, Systemic Inflammatory Response Syndrome; NEWS, National Early Warning Score; MEWS, Modified Early Warning Score; SOFA, Sequential Organ Failure Assessment; UVA, Universal Vital Assessment; ROC, Receiver Operating Characteristic; AUC, Area Under Curve; AUROC, Area Under Receiver Operating Characteristic Curve; LMIC, Low- and Middle-Income Country.

The search was conducted from database inception to June 2025.
