# Supplementary material for: Sepsis screening tools in resource-limited settings: a systematic review and meta-analysis of diagnostic accuracy in low- and middle-income countries
Source: Front Public Health. 2026 May 20;14:1782420. doi: 10.3389/fpubh.2026.1782420 (PMC13230155; doi:10.3389/fpubh.2026.1782420)
Supplement: Supplementary Table S2 — Supplementary analysis of study characteristics for the two continental study populations (Sub-Saharan Africa vs. Asia), supporting the meta-regression analysis and continental subgroup comparisons of qSOFA diagnostic accuracy reported in Section 3.5 (Subgroup analyses). [file Table_2.DOCX]

**Supplementary Table S2.** Comparison of study-level characteristics between African and Asian cohorts included in the systematic review and meta-analysis.

| **Characteristic** | **African Cohort**  **(n = 10)** | **Asian Cohort**  **(n = 15)** |
| --- | --- | --- |
| ***Study characteristics*** | | |
| Number of studies | 10 | 15 |
| Countries represented | Uganda (1), Tanzania (2),  Ethiopia (2), Malawi (2),  Rwanda (2), multi-country  SSA (1)^a^ | India (6), Pakistan (2),  Nepal (1), Sri Lanka (1),  Indonesia (2), Vietnam (1),  Thailand (1), multi-country  SEA (1)^b^ |
| Total patients, n | 10,321 | 12,853 |
| Median sample size (IQR) | 469 (317–1,069) | 387 (196–1,135) |
| Publication year range | 2015–2025 | 2018–2025 |
| ***Study design^e^*** | | |
| Prospective cohort, n (%) | 7 (70.0) | 9 (60.0) |
| Retrospective cohort, n (%) | 3 (30.0) | 6 (40.0) |
| ***Clinical setting^c^*** | | |
| Emergency department, n (%) | 9 (90.0) | 12 (80.0) |
| Intensive care unit, n (%) | 1 (10.0) | 4 (26.7) |
| General ward, n (%) | 4 (40.0) | 2 (13.3) |
| ***Screening tools evaluated^c^*** | | |
| qSOFA, n (%) | 9 (90.0) | 10 (66.7) |
| SOFA, n (%) | 2 (20.0) | 9 (60.0) |
| SIRS, n (%) | 1 (10.0) | 2 (13.3) |
| NEWS/NEWS2, n (%) | 1 (10.0) | 3 (20.0) |
| MEWS, n (%) | 5 (50.0) | 1 (6.7) |
| UVA, n (%) | 3 (30.0) | 0 (0.0) |
| ***Reference standard^c^,d,f*** | | |
| Sepsis-3 criteria, n (%) | 4 (40.0) | 9 (60.0) |
| Clinical diagnosis, n (%) | 3 (30.0) | 2 (13.3) |
| In-hospital mortality, n (%) | 6 (60.0) | 7 (46.7) |
| ***Epidemiological profile*** | | |
| Predominant infection types | HIV-related infections,  tuberculosis, malaria,  bacterial bloodstream  infections | Dengue, typhoid fever,  leptospirosis, community-  acquired pneumonia, urinary  tract infections |
| Key antimicrobial resistance  concerns | Extensively drug-resistant  tuberculosis; emerging  Gram-negative resistance | ESBL-producing  Enterobacteriaceae;  carbapenem-resistant  organisms |
| ***qSOFA diagnostic accuracy (pooled estimates)*** | | |
| Pooled sensitivity (95% CI) | 0.47 (0.36–0.58) | 0.53 (0.44–0.62) |
| Pooled specificity (95% CI) | 0.84 (0.77–0.89) | 0.81 (0.74–0.86) |
| AUROC (95% CI)^g^ | 0.75 (0.71–0.79) | 0.73 (0.69–0.77) |
| Between-group difference (P value) | P = 0.35 (not statistically significant) | |
| Meta-regression: continent as predictor | Coefficient = 0.06 (95% CI: −0.08 to 0.20), P = 0.39 | |

^a^ Moore et al. (2017) was a multi-site study conducted across six sub-Saharan African countries.

^b^ Lie et al. (2018) conducted across Indonesia, Thailand, and Vietnam.

^c^ Studies may contribute to multiple categories (e.g., a study conducted across multiple settings or evaluating multiple screening tools); percentages may exceed 100%.

^d^ Studies may employ multiple reference standards; some studies used Sepsis-3 criteria for sepsis diagnosis while also reporting in-hospital mortality as a secondary outcome.

^e^ Study design classification (prospective vs. retrospective) was determined based on information reported in the original publications and may require verification against individual study protocols.

^f^ Reference standard classification was inferred from the primary outcomes reported in each study; categories are not mutually exclusive.

^g^ AUROC for the African cohort corresponds to the Sub-Saharan Africa subgroup from Table 3. AUROC for the Asian cohort corresponds to the South Asia subgroup from Table 3; a combined South Asia + Southeast Asia AUROC was not separately pooled in the main analysis.

**Abbreviations:** qSOFA, quick Sequential Organ Failure Assessment; SOFA, Sequential Organ Failure Assessment; SIRS, Systemic Inflammatory Response Syndrome; NEWS, National Early Warning Score; MEWS, Modified Early Warning Score; UVA, Universal Vital Assessment; AUROC, area under the receiver operating characteristic curve; CI, confidence interval; IQR, interquartile range; ESBL, extended-spectrum beta-lactamase; SSA, sub-Saharan Africa; SEA, Southeast Asia.

**Note:** Two multi-regional studies (Blair et al. 2023; Rudd et al. 2018) conducted across both African and Asian settings were excluded from this continental comparison to avoid double-counting. The pooled qSOFA diagnostic accuracy estimates for sensitivity and specificity are derived from the direct continental comparison reported in Lines 344–351 of the main manuscript; the between-group P value and meta-regression results are from the same analysis.
